# Maximizing japonica rice quality by high-pressure steam: Insights into improvement

**DOI:** 10.1016/j.fochx.2025.102212

**Published:** 2025-01-23

**Authors:** Saadia Zainab, Xianqing Zhou, Yurong Zhang, Saira Tanweer, Tariq Mehmood

**Affiliations:** a School of Food and Strategic Reserves, College of Food Science and Engineering, Henan University of Technology, Zhengzhou, China; b Department of Food Science and Technology, Faculty of Agriculture and Environment, The Islamia University of Bahawalpur, Pakistan; c Institue of Food Science and Technology, Faculty of Natural and Applied Sciences, Khwaja Fareed University of Engineering and Information Technology, Rahim Yar Khan, Pakistan

**Keywords:** Texture, Viscoelasticity, Mathematical approach, Escalation, Depiction

## Abstract

The research was carried out to optimize the cooking process based on the quality parameters of rice. The steaming process with high pressure proved to have more acceptance among consumers. Based on the textural and gel properties of steamed rice, rice samples soaked for 15 min with a 1:1.3 ratio and steamed at 110 °C and 0.042 MPa were found to be a more effective treatment. Stress-relaxation study and pasting curve model were conducted to comprehensively explain the combinational effect of independent variables. Principal component analysis was carried out to investigate the association between textural and gel characteristics of cooked rice treatments. Characterization was done to check the impact of high-pressure steam on rice. Starch, protein, and amylose content of rice sample were recorded as 78.6, 6.6, and 18.7 % accordingly. High-pressure steam increases the solubility and swelling power while reducing the gelatinization temperature and causing a yellowish color. Pseudoplastic fluid behavior was recorded for both treatments while crystalline structure converted into V-type from A-type in the optimized sample. Optimized samples had lower values of To, Tp, and Tc while higher values of enthalpy and A_21_, A_22_, and A_23_ representing better moisture distribution by breakdown of microstructure obstacles resulting in soft and thick cooked rice.

## Highlights

• High-pressure steaming induces yellowish color.
• High-pressure steam reduces inner moisture bondages.
• High-pressure steam caused V-type structure of cooked rice.
• High-pressure steamed rice exhibits pseudoplastic fluid properties.

## 1 Introduction

Rice is regarded as one of the principal staple diets all over the world (Gao et al., 2024). Consequently, it is one of the abundantly grown cereals with significance prominence from the economic and cultural point of view (Meza et al., 2024). Half of the world's population consumes rice daily and around three-quarters of among acquires 20 % of daily energy prerequisites from rice. Rice grains can be eaten as white rice (polished) or brown rice (unpolished) depending upon the choice of the consumer (Chaturvedi & Manickavasagan, 2024).

China is the largest country producing japonica rice (Ma et al., 2024) where rice cooking methods are associated with the cultural context of the consumers. Normal cooking and pressure cooking in rice cookers are employed in China as household cooking approaches. From the literature pressure cooking proved to be better as it results in improved taste, cuts down cooking time, and lessens perilous elements (Yu et al., 2021). It is evident from the literature that soaking and cooking conditions like soaking ratio, cooking time, cooking pressure, and cooking temperature can significantly affect the micromorphology, texture, and sensory attributes of cooked rice (Xu et al., 2019).

Globally, various methods can be used for the cooking of rice such as the conventional method, the use of rice cookers, and steaming. In the steaming process, rice is cleaned, washed, and soaked normally for 30 min, steamed in a steamer, and hold time is provided at the end. Steaming is a simple process that can lessen the water loss during the cooking of rice and is favorable to developing the special aroma of cooked rice. In the rice cooker, boiling is done either at atmospheric pressure or by providing the pressure by the rice cooker. As electrical rice cookers can regulate temperature and pressure efficiently, therefore they're considered better than that atmospheric cooking as they can also sustain the aroma (Lina & Min, 2022).

High-pressure steaming is gaining quite a lot of attention nowadays in the food sector owing to its several benefits and the consumers' perspectives. High-pressure steam tremendously improved the sensory attributes of the steamed rice. Millard reaction induced by this can improve the aroma and lower the whiteness, making it more appealing to the customers. Moreover, high-pressure steam made the rice softer and sticker by increasing the adhesiveness and reducing the hardness which can be associated with the destruction of starch-protein network and more even water distributions. Even moisture distributions help in the reduction in cooking time and significantly enhance the taste quality of cooked rice. High-pressure steaming is a fast approach and consistent quality is achieved at the industrial level (Xu et al., 2019). It also guarantees the equal distribution of heat while heating the food and minimizes nutrient losses. Convincedly, it is an auspicious approach to encounter the essentiality to promote the shelf life and enhance the taste quality of cooked rice (Zhang et al., 2024). So far studies have been frequently focused on the rice cooked by conventional approach or rice cooker. There is insufficient research conducted on the high-pressure steam used for the cooking of rice. So, the aim of the research is to optimize the high-pressure steaming process and evaluate the results by comparing it with the control sample. In this research steaming with no pressure (at the atmospheric level) as a control treatment was compared with high pressure steaming process to investigate the impact of high-pressure steam on the main characteristics of the rice. But before that high-pressure steaming process was improved by using the surface response method in terms of textural and gel properties (eating qualities). In which textural and gel parameters were recorded against various combinations of chosen independent variables and analyzed. This study makes major contributions to the said theme. Firstly, the high-pressure steaming process is optimized by using the RSM approach. Secondly, the stress relaxation test and pasting curve modeling explained the effect of each independent variable in detail. Thirdly, the optimized rice sample is compared with the control sample to explore the main alterations caused by the high-pressure steam process.

## 2 Materials & methods

### 2.1 Materials

Long-grain incense (Japonica rice, Chalinhe Farm, Harbin City, Heilongjiang Province) was used for this study. Sodium acetate, sulfuric acid, and sodium hydroxide were purchased from Shanghai Aladdin Biochemical Technology Co., Ltd.

The instruments used for the research are: Rice appearance quality tester (Zhejiang Bethlehem Instrument & Equipment Co., Ltd.), Intelligent Pressure Steam Room (Pingdingshan Baiyangfeng Machinery Manufacturing Co., Ltd.), TA.XT Plus Texture Analyzer (Stable Micro Systems, UK), RVA-TecMaster Rapid Viscosity Analyzer (Australia Botong Company), 101 Electric Blast Drying Oven (Beijing Guangming Medical Instrument Co., Ltd.), MICGIA type Calorimeter (Satake Company, Japan), LXJ-IIB Low-speed and Large-capacity Centrifuge (Shanghai Anting Instrument Factory), Differential Scanning Calorimeter (DSC, TA Instruments, New Castle, DE, USA), RS6000 Hack Dynamic Rheometer (Thermo Fisher Technology Co., Ltd.), D8 Advance X-ray diffractometer (Bruker, Germany), MicroMR-CL-1 Variable Temperature Nuclear Magnetic Resonance Food Agricultural Imaging Analyzer (Shanghai Newman Electronic Technology Co., Ltd.).

### 2.2 Appearance test

A rice appearance quality tester was used for appearance analysis. Rice length-to-width ratio and breakage rate were determined (Lu et al., 2023).

### 2.3 Physicochemical determination

AOAC (2006) and AACC (2015) international standards were adopted for the determination of physicochemical properties. For the assessment of moisture content, ash, starch, and amylose content AACC method 44–01.01, 08–01.01, 76–11.01, and 61–03.01 were followed respectively. While AOAC methods 930.29 and 920.39 were employed for the estimation of crude protein and crude fat accordingly.

### 2.4 Experimental design

Design expert (Version 13, Minneapolis, USA) was used for the experimental design. Response surface method (RSM) optimization was carried out by choosing a level three-factor Box-Behnken Design (BBD). The independent variables chosen were steaming conditions (Temperature: Pressure) (X_1_), Soaking time of rice (X_2_), and Rice to water ratio (X_3_). Independent variables with their levels are exhibited in Table S1. The levels of independent variables were chosen based on the preliminary conducted experiments. The resultant design of 17 experimental runs generated by RSM BBD is described in Table S2.

### 2.5 Rice steaming process

**Fig. 1:**
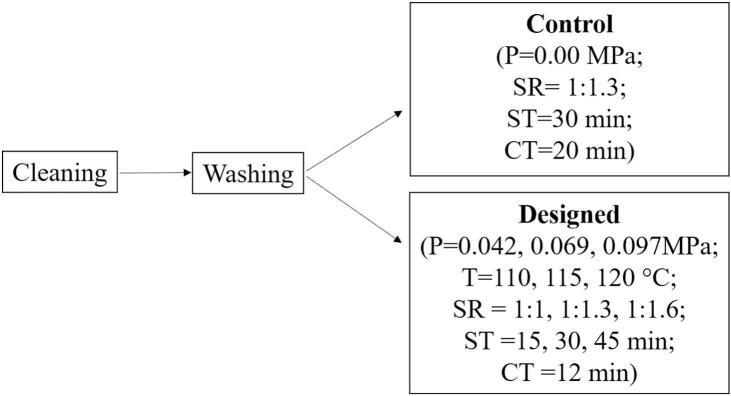
Selection of Independent Variables for the research.

An intelligent pressurized steamer was used for the steaming of rice. Xu et al. (2019) method with slight modification was adopted for the steaming process. Before the steaming process, rice samples were cleaned, washed, and soaked. The soaking ratio and soaking time used were 1:1.3 and 30 min respectively for both the steamer and control study. The samples were steamed under different pressure for 10 min and 2 min hold time was provided. The controlled sample was steamed by using an induction plate to steam the rice sample for 20 min. Temperature, pressure, and soaking ratio were selected as independent variables which are depicted in Fig. 1.

### 2.6 Texture profile analysis

#### 2.6.1 Determination of TPA parameters

Textural characteristics of steamed rice samples were determined by following the method of Ha et al. (2024) with slight modifications. After steaming, rice samples were cooled down to room temperature to conduct the texture profile analysis. Rice samples were compressed twice to 50 % by using a P/36R probe. The pre-test, test, and post-test speeds were adjusted to 2 mm/s, 1 mm/s and 2 mm/s respectively. The trigger force used was 5 gf.

#### 2.6.2 Stress relaxation study

Viscoelastic properties of the gels were determined through a stress relaxation test. The gels from RVA were directly subjected to the test after cooling them down to room temperature. The gel samples were compressed to 50 % deformation and allowed to relax for 120 s. The pre-test, test, and post-test speeds were 10 mm/s, 2 mm/s, and 5 mm/s accordingly. The relaxation curves were initially normalized by using Peleg and Normand's Eqs. (1), (2) (Kadival et al., 2023).

(1) $$Y\left(t\right)=\frac{F_{0}-F_{t}}{F_{0}}\;$$

(2) $$\frac{t}{Y}=\frac{1}{\mathrm{ab}}+\frac{t}{a}=k_{1}+k_{2}t$$

where; Y(t) = decaying parameter;

F_0_ = Force at the beginning;

F_t_ = Force at any moment t;

k_1_ and k_2_ = intercept and slope of the graph plotted between t/Y and t;

a and b = constants exhibiting the extent of stress decay and rate of stress decay.

Residual force or equilibrium force when t → ∞ was estimated by employing the Eq. (3)

(3) $$F_{\infty }=F_{0}-\frac{F_{0}}{k_{2}}$$

### 2.7 Pasting properties

#### 2.7.1 Rapid visco analyzer

Pasting properties were determined by using the rapid visco analyzer (RVA) on a 14 % moisture basis. The method adopted was explained by Zainab et al. (2024) with minor modifications. The slurry of rice flour was prepared by adding 3 g of rice flour in 25 g of water in an aluminum canister which was mixed properly with the help of a plastic peddle to make sure no lump formed. The temperature was balanced at 50 °C for 1 min and the paddle speed was initially set at 960 rpm which was changed to 160 rpm after 10 s. Temperature increased from 50 to 95 °C in 3 min 45 s and sustained for 2.5 min. The sample was again cooled down up to 50 °C in 4 min and ended in 1.5 min. Values of the peak, trough, final, breakdown, and setback viscosities were recorded.

#### 2.7.2 Pasting curve model construction

The pasting curves acquired from RVA were subjected to the first-level kinetic equations. For the analysis, the RVA curve is cleaved into 4 conspicuous divisions each of which exhibits a more thorough examination of the RVA data (Zhang et al., 2023).

Part 1: This part is from the commencement of the pasting curve to the peak viscosity with a constant shear rate (160 rpm) and continuously increasing temperature. Hill model (Eq. 4) is applied to acquire the fit data.

(4) $$\mathrm{Visc}=\frac{v_{\mathrm{peak}}\;x\;t^{s}}{t_{50}^{s}+t^{s}}$$

where, Visc = Apparent viscosity (cP).

$v_{\mathrm{peak}}$ = Peak Viscosity (cP).

t= Time (s).

$t_{50}$= Time to attain 50 % of peak viscosity.

s= Countenance of the dissolution rate starch coefficient (constant).

**Fig. 2:**
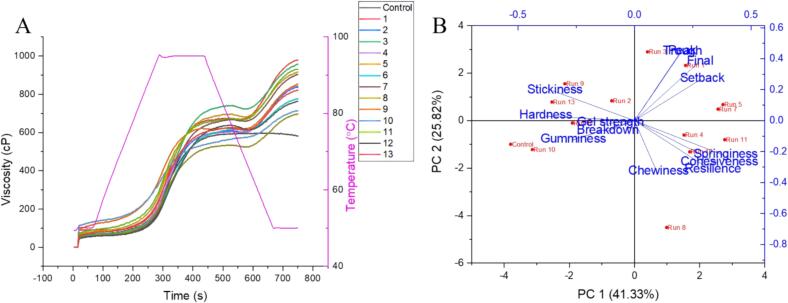
**A:** RVA Curves, **B:** Principal component analysis of TPA and RVA parameters of steamed rice.

Part 2: It starts from the peak viscosity and concludes when the temperature reaches a constant value of 50 °C which is observably absent in pasting curves of steamed rice samples that can also be seen from Fig. 2A. Generally, the Arrhenius Eq. is used for this part (heating process).

Part 3: At constant temperature (95 °C) and speed (160 rpm), the association between time and viscosity is modeled by using Eq. 5.

(5) $$\mathrm{Visc}=K\exp\left(-\mathrm{nt}\right)$$

where, Visc = Apparent viscosity (cP).

K = Consistency coefficient (cP).

n = Fluid behavior index (s^−1^).

t = Processing time (s).

Part 4: It consists of the valley viscosity beginning to the point until the temperature drops to the constant value of 50 °C. Arrhenius Eq. (6) is employed to determine the relationship between viscosity and time.

(6) $$\mathrm{Visc}=A_{0}\exp\left(\frac{E_{a}}{\mathrm{RT}}\right)$$

where,Visc = Apparent viscosity (cP).

$A_{0}$ = Prefactor model constant (cPa).

$E_{a}$ = Activation energy (kJ.mol^−1^).

R = Molar gas constant (8.314 J.mol^−1^).

T = Temperature (K)

### 2.8 Gel strength

The gel strength of the samples was determined by adopting the Hu et al. (2020) method with slight modifications. Samples were cooled down to room temperature before conducting the test. A cylindric probe was used with a test speed of 0.8 mm/s and 5 g trigger force.

### 2.9 Characteristics of the optimized sample

#### 2.9.1 Color

For the color determination of steamed rice, instant rice, and rehydrated rice, 10 points on a Petri plate (90 mm) filled with rice were selected randomly. L*, a*, and b* were recorded. The measurements were made in triplicates and were expressed as averages. L* is the measure of brightness from black (0) to white (100), a* describes red-green color where +a value exhibits redness and -a values exhibit greenness while b* describes yellow-blue color where +b represents yellowness and -b exhibits blueness (Devi & Badwaik, 2022).

#### 2.9.2 Thermal properties

Thermal properties were determined by using DSC by improvising the method explained by Farooq et al. (2021). Peak (Tp), onset (To), conclusion (Tc) temperatures, and enthalpy of gelatinization (∆H) were recorded.

#### 2.9.3 Solubility & swelling power

The solubility and swelling power of cooked rice samples were determined by following the method explained by Yang, Hao, et al. (2021).

#### 2.9.4 Rheological properties

Rheological properties were appraised by Kadival et al. (2023).

##### 2.9.4.1 Steady-state rheological measurements

The steady-state rheological measurements were carried out in the shear rate range of 0–250 s^−1^. The sample flow behavior was modeled using the power law (Eq. 7).

(7) $$\tau =K{\gamma^{.}}^{n}$$

where τ = Shear stress in Pa;

K = Consistency index in Pa.s^n^;

$\gamma^{.}$ = Shear rate in s^−1^;

n = Flow behavior index.

##### 2.9.4.2 Dynamic rheological measurements

Sweep tests were conducted at a constant frequency (0.1 rad·s-1) with varying shear strain (0.1–100 %). The power law model was applied to the data obtained to observe the frequency dependency of $G^{\prime }$and $G^{\prime \prime }$ (Eq. (9), (10)).

(9) $$G^{\prime }=K^{\prime }{\left(\omega \right)}^{n}$$

(10) $$G^{\prime \prime }=K^{\prime \prime }{\left(\omega \right)}^{n}$$

where $G^{\prime }$ = Storage modulus in Pa;

$G^{\prime \prime }$ = Loss modulus in Pa;

ω = Frequency in Hz;

n${}^{\prime }$ and n${}^{\prime \prime }$ = Power law exponents;

K${}^{\prime }$ and K${}^{\prime \prime }$ are constants.

From the values of storage and loss modulus, the complex modulus was also calculated by using Eq. (11).

(11) $$G_{*}=\surd {\left(G^{\prime }\right)}^{2}+{\left(G^{\prime \prime }\right)}^{2}$$

#### 2.9.5 Crystalline structure

The crystalline structure of the steamed rice was determined by following the method of Bae et al. (2023) and the crystallinity of the samples was assessed.

#### 2.9.6 Water distributions

NMR analysis was carried out for the assessment of alteration in water transportation. After slight modifications, the Witek et al. (2020) method was followed.

### 2.10 Statistical analysis

To compare the mean values of treatments, the results obtained were subject to the one-way ANOVA and Tukey's test by using IBM SPSS Statistics 2023 and for graphical representation Origin, 2023 was used. Partial component analysis (Singular value decomposition) was used to lessen the extent of analyzed parameters of TPA and RVA and to establish the linkage among these parameters.

## 3 Results and discussions

### 3.1 Appearance and physicochemical tests

The recorded parameters of the appearance test and physicochemical are depicted in Table S3. The appearance of rice plays a major role in acquiring the attention of consumers. Consumers consider a lot about the length, Chalkiness, broken rate, and color of the rice while consuming and associate them with the quality of rice. Chemical and physical characteristics play a vital role in determining the rice quality (Hu et al., 2022). Starch, protein, and fat content significantly affect the rice quality. Starch being a major constituent determines the gelatinization and cooking properties of the rice. Proteins, being 2nd major contributor to rice, also influence most of the quality properties of cooked rice (He et al., 2021). Starch, protein, and amylose recorded values were 78.6, 6.6 and 18.7 % respectively. Starch with protein produces a complicated network and influences many rice characteristics (Xu et al., 2019).

### 3.2 Selection of levels of independent variables

The steaming process combination of temperature and pressure was determined from the following graph (Fig. S1). Soaking time and soaking ratio were selected through experiments and published literature. Temperature levels (100, 115, 120 °C), pressure levels (0.042, 0.069, 0.097 MPa), soaking time levels (15, 30, 45 min), and soaking ratio levels (1,1, 1:1.3, 1:1.6) were used for the said study.

### 3.3 Effect of steaming conditions on textural properties of cooked rice

#### 3.3.1 TPA of rice & gel strength

The textural properties of cooked rice and gel strength are portrayed in Table S4. Range of hardness, stickiness, chewiness, gumminess, springiness, cohesion, resilience, and gel strength varies from 6555 ± 156 to 1135 ± 107, 1026 ± 22 to 137 ± 17, 1247 ± 183 to 275 ± 26, 1943 ± 102 to 533 ± 40, 0.76 ± 0.08 to 0.27 ± 0.01, 0.62 ± 0.02 to 0.26 ± 0.01, 0.40 ± 0.03 to 0.07 ± 0.01, and 50.2 ± 2.51 to 24.53 ± 1.22 respectively for the experimental design prepared.

It is clear from the data that not only temperature and pressure impart the textural characteristics of the rice samples but soaking time and soaking ratio also play key roles in determining the textural attributes of the rice. For runs 1,2, and 3 although the same temperature and pressure (120 °C; 0.097 MPa) but due to various soaking conditions different values were obtained among which run 3 (1:1 & 30 min) had higher values of hardness, stickiness, chewiness, gumminess, and gel strength. Similarly among runs 4–7 (115 °C; 0.069 MPa), 8–9 (110 °C; 0.042 MPa), and11–12 (110 °C; 0.042 MPa), run 6 (1:1 & 15 min), run 9 (1:1.6 & 30 min), and run 11 (1:1.3 & 45 min) had the higher recorded values accordingly. Among all the runs, 13 (115C, 0.069) had a slightly different trend and had comparatively higher values of only hardness and stickiness which can be attributed to the soaking conditions of (1:1.3 & 30 min). These outcomes identified the impact of independent variables on the textural parameters of high-pressure steamed rice.

#### 3.3.2 Stress-relaxation test

Stress relaxation curves of the designed experimental design are corroborated in Fig. S2. As time increases, stress values reduce until they reach a limited equilibrium value (F∞ *>* 0). This type of action is the characteristic of viscoelastic solid materials depicting disruptions of weak bonds while strong bonds remain unaffected by this deformation progression. The Peleg model was applied to the stress relaxation data. The model parameters F_o_, F_ꚙ_, k_1_, k_2_, a, b, R^2^ are provided in Table 1. F_o_, F_ꚙ_ follows the same trend as textural characteristics. Elevated values of k_1_ illustrate lower relaxation representing stronger and more flexible gel. k_1_ values are exact in accordance with that of gel strength obtained from the texture analyzer shown in Table 1. Protein with starch form a complex network and compete for the water for swelling hindering the gelatinization process (Kadival et al., 2023) which impacts the gel strength and flexibility. While the fitness of the model on data can be perceived by R^2^ values.

**Table 1:** Stress relaxation model parameters.

Samples	F_o_ (N)	F_ꚙ_ (N)	k_1_	k_2_	a	b	R^2^
**Control**	0.01911	0.018714	45.58	48.27	0.020716	1.05899	0.999
**1**	0.01795	0.01783	815.81	150.12	0.006661	0.184013	0.927
**2**	0.03959	0.039337	706.82	156.68	0.006382	0.221669	0.979
**3**	0.02399	0.023831	1053.70	150.86	0.006629	0.143172	0.980
**4**	0.03203	0.031425	85.63	52.94	0.018889	0.618263	0.998
**5**	0.03005	0.029682	318.20	81.56	0.012261	0.256307	0.996
**6**	0.02209	0.02186	180.72	96.19	0.010396	0.532249	0.994
**7**	0.02754	0.02712	97.67	65.53	0.015261	0.670933	0.998
**8**	0.02587	0.025714	810.97	165.76	0.006033	0.204397	0.987
**9**	0.02811	0.027574	166.57	52.46	0.019063	0.314931	0.998
**10**	0.02959	0.029399	540.97	155.26	0.006441	0.287003	0.998
**11**	0.03016	0.029653	49.10	59.49	0.01681	1.211474	0.999
**12**	0.03185	0.031739	2077.30	286.42	0.003491	0.137881	0.820
**13**	0.03955	0.03945	5359.20	396.17	0.002524	0.073923	0.787

**Where**; F_o_ and F_ꚙ_ are forces at the initial and equilibrium stages. k_1_ and k_2_ are the intercept and slope of Peleg's model. ‘a’ and ‘b’ are the constants that explain the extent of stress decay and rate of stress decay accordingly.

### 3.4 Effect of steaming conditions on pasting properties of cooked rice

#### 3.4.1 RVA analysis

RVA recorded the changes in viscosity over time as temperature changes to provide insights into the gelatinization process. The gelatinization process of starch mainly involves the water absorption, swelling, and destruction of the crystalline arrangement resulting in the percolation of AC. Gelatinization temperature and pasting viscosity can be mainly affected by the swelling power, water edging capacity, shear defiance, and contest for the water between leached amylose and unhydrolyzed starch granules (Song et al., 2024).

14 curves were obtained (Fig. 2A, Table S5) depicting the pasting process of various combinations of steaming and soaking conditions. Almost all combinations followed the same standard trend of the curve by initiating the gelatinization process with the increase in temperature where starch molecules started to absorb water and swell up until the peak viscosity was obtained. After that viscosity declines and starch molecules start to break down until a paste of a certain consistency is obtained and the viscosity value starts to increase again representing the retrogradation process where starch molecules again set themselves and rearrange to attain the structure where water entrapped again in starch molecules (Kadival et al., 2023).

Among the groups of the same temperature and pressure combinations, run 3 (120 °C; 0.097 MPa), 6 (115 °C; 0.069 MPa), 9 (110 °C; 0.042 MPa), and 11 (110 °C; 0.042 MPa) had the highest values of pasting parameters which can also be observed by RVA trend depicted in Fig. 2A.While the control sample had quite lower values in comparison to the high-pressure steam treated rice which can be attributed to the promotion of degree of gelatinization by high-pressure steaming. This occurs due to the increased heat transfer and moisture content, leading to starch granules absorbing water, swelling, and rupturing, forming a gel through the leaching process. This process is more pronounced at higher temperatures, leading to a higher degree of gelatinization (Taghinezhad et al., 2016). The more precise information about the gelatinization process can be assessed by modeling pasting curves.

#### 3.4.2 Paste kinetic model parameters

To comprehensively understand the pasting of cooked rice samples, models were used to interpret the data obtained. The range of R^2^ (0.83 to 0.95) explained that all models are moderately good to excellent fit to the data, explaining the pasting process. The first part of the curve represents a sigmoidal manner. In this section, PV is used to interpret the process but how fast PV is achieved, there is no evidence in the published literature although “t_50_” in the model represents the time required to acquire 50 % of the peak viscosity. The other dimensionless parameter “s” exhibits the extent of swelling. s > 1 corresponds to the enhanced starch granule swelling rate by penetration of water while s < 1 can be associated with the declined starch granule swelling rate by incorporation of water (Song et al., 2024). For all the runs, s > 1 indicates improved grain swelling as high-pressure steaming disrupts the starch structure and makes it easy for water to penetrate.

During gelatinization, 2 step progression works simultaneously i.e., hydrogen bonds breakage in the amorphous region of starch and water absorption and enlargement of these areas. Referring to Table 2, runs 3 and 7 have 2.65, and runs 4 and 8 have 2.42 “s” values. The overlapping and same “s” values can be seen against different t_50_ values which can be associated with the stronger hydrogen bonds under the same hydration and swelling of starch in amorphous portions. “s” value can be related to the potential factors that are different soaking and steaming conditions provided which can affect gelatinization process by examining the same starch at same heating rate.

**Table 2:** Modeling parameters assessed from pasting behavior.

	Part 1				Part 3			Part 4		
Samples	*v*_peak_ (cP)	t_50_ (s)	s	R^2^	K (cP)	n*10^3^	R^2^	A_0_ (cP)	E_a_ (kJ/mol)	R^2^
**Control**	285.71	289.06	2.38	0.90	140.42	3.3	0.95	5.66	2192.90	0.83
**1**	370.37	308.16	2.01	0.85	186.8	3	0.92	1.09	18,550.20	0.91
**2**	263.16	308.33	2.65	0.86	40.24	6.5	0.88	1.51	16,802.59	0.88
**3**	357.14	315.16	2.18	0.86	31.36	7.6	0.92	3.34	15,039.19	0.87
**4**	303.03	303.22	2.42	0.87	59.34	5.6	0.87	1.87	16,237.24	0.89
**5**	500	300.22	1.72	0.89	67.32	5.6	0.91	2.75	15,442.42	0.88
**6**	303.03	312.66	2.6	0.85	19.9	8.4	0.95	7.19	12,432.76	0.86
**7**	277.78	314.47	2.65	0.85	80.94	4.9	0.93	2.26	15,908.01	0.87
**8**	333.33	300.87	2.42	0.87	51.94	5.6	0.89	2.16	15,366.77	0.88
**9**	1666.67	274	1.05	0.90	155.21	3.4	0.85	1.78	16,403.52	0.88
**10**	2000	255.67	0.88	0.89	161.91	2.9	0.89	10.42	11,228.89	0.87
**11**	666.67	292.87	1.48	0.90	90.22	4.8	0.88	1.68	16,800.93	0.90
**12**	322.58	317	2.57	0.84	25.35	7.6	0.92	7.30	12,355.44	0.84
**13**	454.54	298.5	1.87	0.88	66.73	5.3	0.89	2.48	15,431.62	0.87

Gels were sheared continuously in the third region at a constant temperature of 95 °C. The declining trend of data was fitted to the exponential model and relevant parameters are enlisted in Table 2. As R^2^ values range between 0.85 and 0.95 the chosen model had satisfactorily explained the association of time and viscosity. Viscosity in this section is entirely associated with damaged starch. Damaged starch is directly proportional to the viscosity value in this section. The values obtained directly exhibit the interior formation of the granule and its technical characteristics. Higher the viscosity value represents the brittle construction of gels against shearing. So, it can be concluded that, against shear, damaged starch content plays an important role in gel stability. The stability of starches decreases with the increment of damaged starch content. This feature can also help in improving the process manufacturing of rice starch by enhancing the rice gel stability against shear.

In the 4th part, rice starch gels cooled down from 95 to 50 °C and the data obtained was fitted to the Arrhenius equation and the model parameters can be seen in Table 2. R^2^ value varies from 0.83 to 0.91, explaining the good fit of the model to interpret the gel behavior in cooling mode. Ea values fall in the range of 2192.90 to 18,550.20 kJ/mol. Ea values of runs 1, 2, 4, 9, and 11 are comparatively higher which aligns with the trend obtained for setback values. In the cooling process, amylose leached from the starch granule during gelatinization formed a solid structure by interrelating the with amylopectin sequences of swelled starch and elevating the firmness of cooked rice because of recrystallization of starch molecules. So, Ea values of the Arrhenius equation of this part can be interpreted as the retrogradation rate. Elevated values of Ea represent the increased retrogradation rate that can be related to the free amylose content not bound to the lipids. Normally in literature, setback value is used for the comparison and interpretation of the starch retrogradation rates (Palabiyik et al., 2017).

### 3.5 PCA

The principal component analysis is a widely used method (Zhang et al., 2019) that comprehensively gives the idea of association among analyzed parameters. It can help in highlighting the comprehensive indicators and assess their corresponding momentousness along with the maintenance of prominent information after dimensionality reduction at a convincingly elevated degree (Shi et al., 2021). To explore the textural and pasting properties of high-pressure steamed rice, data was analyzed by PCA. From Table S6, the principal components selected whose eigenvalues were above 1.0, and the cumulative rate of those became 89.75 % exhibit heaps of information regarding the cooked rice. It can be seen that 4 principal components can be used for the interpretation of evaluation of the cooking phenomenon of rice based on the steaming conditions, soaking ratio, and time. The first, second, third, and fourth principal components contribute to the 41.33, 25.82, 14.26, and 8.34 % respectively to make the accumulating variance of 89.75 %. The biplot obtained from the PCA is shown in Fig. 2B. The biplot explains the 14 treatments and eigenvalues of principal components. The first PC compromises springiness, cohesiveness, resilience, hardness, stickiness, gumminess, gel strength, and breakdown while the second one compromises chewiness, peak viscosity, trough viscosity, final viscosity, and setback.

### 3.6 Process optimization

#### 3.6.1 Fitting of model

Response Surface Methodology (RSM) is a mathematical and statistical approach widely used for the modeling and analysis of problems. In this approach process is optimized through experimental design and empirical models for the response prediction as well as operation of anticipated function for multiple response optimization (Weremfo et al., 2022). The most appropriate response values were recorded by providing the steaming conditions of 110 °C and 0.042 MPa, soaking ratio of 1:1.3 for 15 min. In the whole process of optimization, linear, second-order, and quadratic models were used for the description of recorded responses which all are explained in Table S7 regardless of the low fitness of the model to interpret the results more smoothly and comprehensively.

#### 3.6.2 Achieving optimum conditions

To have a clearer impression of the effect of steaming temperature, steaming pressure, soaking ratio, and soaking time on the whole scenario, 3D graphs drawn by design expert software are shown against each response parameter. Through these graphs' values of each response, either increment or reduction within the range of independent variables are obvious and aligned with the data obtained by TPA and RVA. Also, run 8 is the optimized condition after analyzing all the response parameters. The best-fitted response surface is expressed in Fig. S3 & S4.

#### 3.6.3 Verification of model

After implementing the models, optimized conditions were reassessed to check the goodness of fitness of the model regarding the prediction of response parameters' values. Optimized steaming and soaking conditions were validated by steaming the rice at 110 °C and 0.0042 MPa and soaking the rice for 15 min by using the ratio of 1:1.3. Hardness, stickiness, springiness, chewiness, gumminess, cohesiveness, resilience, peak viscosity, trough viscosity, breakdown, final viscosity, and setback values of steamed rice recorded were 4002.65, 605.80, 0.6502, 1085.2, 1820.56, 0.5143, 0.3285, 473, 477.5, −3.0853, 682.4, 172.12 accordingly. Extraordinary confirmation among predicted and observed response values proclaims the authentication of models.

### 3.7 Characterization of controlled and optimized conditions

#### 3.7.1 Color

**Fig. 3:**
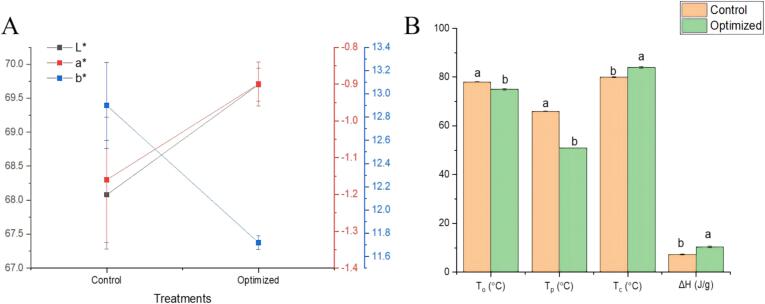
**A:** Colorimetric values, **B:** Thermal properties.

Color is the physicochemical aspect that can be influenced by the steaming conditions and also plays an important role as a quality indicator (Karimidastjerd et al., 2024). Whiteness is the basic perception of the rice's appearance quality. The higher value of whiteness can be linked to the positive perception of customers towards rice quality (Aznan et al., 2023). The calometric values are exhibited in Fig. 3A. The optimized treatment had less whiteness and more yellowness as compared to the control one which can be related to the high-pressure steam. At higher temperatures and pressure, the Maillard reaction occurs due to which the lightness of steamed rice decreases while the yellowness increases (Xu et al., 2019). High temperatures favor 2-acetyl-1-pyrroline depletion compared to its generation favoring the Maillard reaction (Cai et al., 2024). Although there's a difference among the values of L*, a*, and b* of control and optimized rice samples that difference is not that great representing that the optimized high-pressure steamed rice sample doesn't differentiate too much from the control sample in terms of color which can be easily accepted by the consumers owing to many other advantages of high-pressure steaming.

#### 3.7.2 Thermal characteristics

Thermal parameters of control and optimization are depicted in Fig. 3B. Thermal characteristics are used to appraise the crystalline melting comportment of cooked rice. With the high-pressure steam, rice pre-gelatinized and achieved a certain amount of damaged starch hence reducing the gelatinization temperature (Zhu et al., 2020). Comparatively higher gelatinization and lower enthalpy values can be associated with the increment of solubility and swelling (Farooq et al., 2021). The improvement in solubility and swelling power makes it easy for water to be entrapped in the granule, facilitating the gelatinization process. High-pressure steam enhances the amylose leaching during the steaming process causing the lowering of To. High-pressure steam disrupts the starch structure and lowers the gelatinization temperature resulting in the reduction of To, Tp, & Tc. The optimized sample had a higher value of enthalpy change than that of the control sample which can be associated with the destruction of the starch-protein network caused by high-pressure steam leading to better moisture distributions leading to stickier and softer texture (Xu et al., 2019). The improved moisture distribution and proper heat allocation assist in the enhancement of enthalpy change value (Hu et al., 2023).

#### 3.7.3 Rheology

Rheological properties are considered an important feature as they can affect the transportation, hydrolysis, and absorption of hydrolyzed nutrients within the gastrointestinal tract that directly affect the gastric emptying rate. So, the rheology of cooked rice is a detrimental factor to the eating quality of cooked rice (Na-Nakorn et al., 2021).

##### 3.7.3.1 Strain sweep

**Fig. 4:**
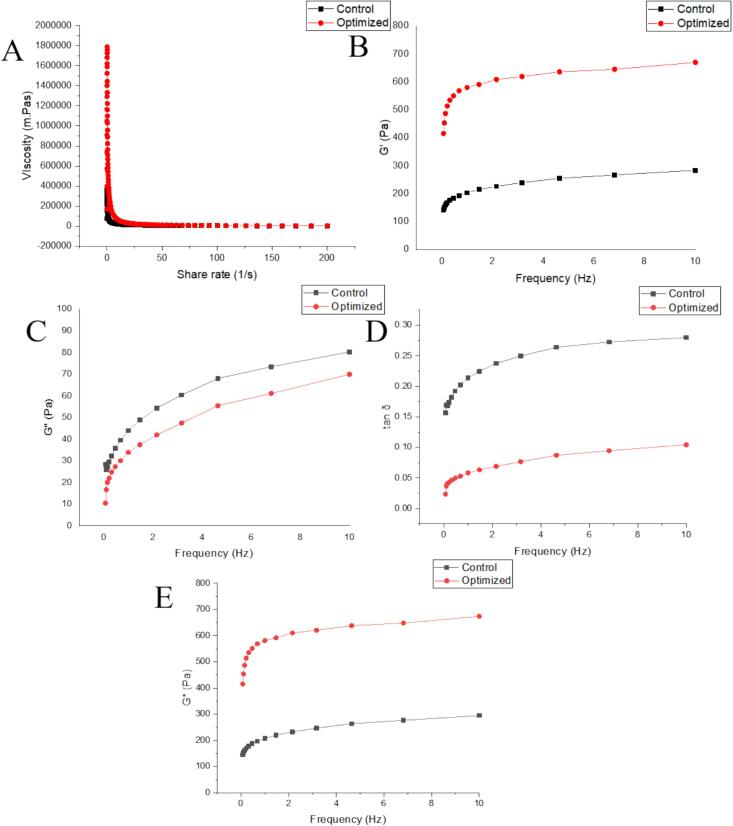
**A:** Apparent viscosity, **B:** Storage modulus (G'), **C:** Loss modulus (G"), **D:** Loss tangent (tan δ), **E:** Complex modulus (G*).

As can be seen from Fig. 4A, the apparent viscosity of the three samples had high values at a low shear rate, and then with the increase of shear rate, the apparent viscosity decreases rapidly. The rapid decrease values fall in the range of shear rate 0–25 s^−1^ and gradually stabilizes when the shear rate reaches up to 50s^−1^, which trend belongs to the shear thinning phenomenon unique to pseudoplastic fluids, that is, the apparent viscosity of the fluid decreases with the increase of shear rate (Hassan et al., 2022). It is often used to express the shear stability of a system during the shear process. The viscosity of the control treatment is less than that of the optimized which could be linked to the more soaking time as it is evident from the literature that more soaking time can lower the amylose content (Shen et al., 2022) hence amylopectin proportion of polymerization increased and caused hindrance to the flow (Yan et al., 2021).

The power law model was applied for further analysis of the shear-thinning manner of the samples. R^2^ values (Table S8) represent the fitness of the model. The values *n* < 1 depict the Non-Newtonion fluids while the K is the flow consistency coefficient representing the thickness or viscosity of fluid i.e., apparent viscosity. With the application of high-pressure steam, the value of “n” reduces depicting the improvement in shear thinning behavior (more viscous paste) while the value of “K” increases. These results can be associated with the amylose leaching during cooking, making the gel samples more viscous which also aligns with the outcomes of gelatinization, pasting properties, solubility, and swelling power.

##### 3.7.3.2 Frequency sweep

The frequency sweep mode is used to reflect the changes in the viscoelastic and network structure of the starch gel structure (Ramli et al., 2022). Elasticity and viscosity of the system can be represented by the storage and loss modulus while the strength of the system can be expressed in terms of complex modulus which is a combined effect of storage and loss modulus. Tanδ is the ratio of viscous to elastic modulus, a higher value represents the viscous nature of the system. Optimized treatment had higher values of G' and G* as compared to the control ones, representing more stability as opposed to distortion (Fig. 4B, E). While the optimized treatment had lower values of G" and tanδ in comparison to the control treatment (Fig. 4C, D). Most variations of tanδ values occurred in the range of 0–0.5 Hz exhibiting the elastic behavior.

The lower value of tanδ indicates more elastic behavior was induced by the high-pressure steaming process. Higher G' and lower G" exhibit firm structure and improved elasticity that portrays a more stable structure subject to stress. The combined effect of higher G' and lower tanδ can be linked to the enhanced quality and palatability of cooked rice. Power model parameters are mentioned in Table S8 where K, n, and R^2^ represent the consistency coefficient, flow index, and goodness of fitness of the model respectively. As all R^2^ > 0.90 correspond to the high fitness degree. For n < 1 values pointed to the pseudoplastic fluids that are consistent with the dynamic viscosity results.

#### 3.7.4 Solubility and swelling power

**Fig. 5:**
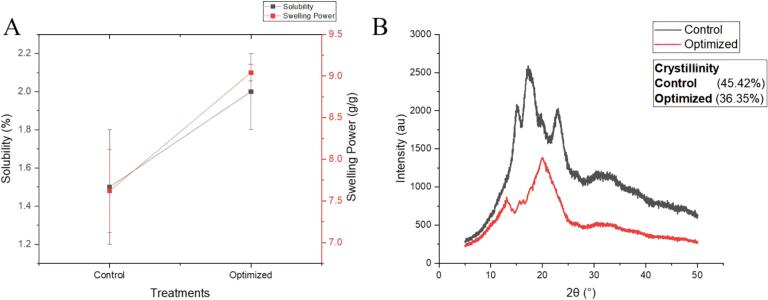
**A:** Solubility and swelling power of steamed rice **B:** XRD pattern of steamed rice.

The solubility and swelling power of starch can be used to evaluate the degree of interaction between starch chains and within the crystalline and amorphous domains and can also reflect the ability of starch to bind water (Kumar et al., 2023). It can be observed from Fig. 5A control treatment had lower values of solubility and swelling power while the optimized sample had higher values. High-pressure steam can interrupt the structure of cooked rice (Xu et al., 2019). High pressure can help ease the leaching of amylose content resulting in enhanced solubility. Solubility increment is directly associated with the high-pressure steam as higher temperature and pressure caused the destruction of cell walls of the granules subsequent to starch releasing in water. Swelling power also increased as high pressure and temperature disrupted the starch granule structure that endorsed the water incursion and enhanced the capability of swelling degree which can also be linked to the higher gelatinization and digestibility. The results of solubility and swelling power aligned with the outcomes of thermal properties, gelatinization, and rheological attributes. Improved swelling power and solubility contribute more to the eating quality of cooked rice by affecting the texture, appearance, and mouthfeel. Enhanced swelling power depicts more expansion of rice grains during steaming leading to a softer and better palatability texture. Higher solubility enables improved flavor release and a more appealing consistency and a less dense structure is achieved that averts grain hardness (Zainab et al., 2024).

#### 3.7.5 Crystallinity

XRD approach provides the vision of the crystallinity of the sample. The molecular structure is of great significance in specifying the hardness and stickiness of cooked rice, assisting rice breeders, industrialists, and consumers to enhance the cooking and eating quality of the rice (Li & Gilbert, 2018). XRD pattern of cooked rice samples is shown in Fig. 5B. Control treatment exhibited 4 peaks while 2 peaks were recorded for the optimized treatment. Peaks of control treatment recorded at 15°, 17°, 18°, and 23° exhibit an A-type crystalline structure (Liu et al., 2020) that could be related to the structural arrangement of rice starch. The constricted structure of starch is formed by the configuration of a double helix with short side chains of amylopectin. The reflections at 2θ of 13° and 20° can be associated with the V-type in the pattern of optimized treatment (Ren et al., 2022). High-pressure steam destroys the protein and starch network in steamed rice, and lipids established complexes with the helical structure of amylose (Xu et al., 2019). While the minute amount of lipids in comparison to protein formed a weak association and represented more amorphous peaks. With the destruction of the native crystal structure in the optimized sample, new single and double helix stacking arrangements appeared by high-pressure steaming process inducing a new V-shaped crystal structure (Li et al., 2024). This induced structure is quite health beneficial as it enhances the starch digestibility and antioxidant activity as well as causes intervention against chronic diseases. V-type of starch acts as dietary fiber as it is much resilient to the digestion by enzyme present in small intestine which is later fermented by gut bacteria in colon resulting in short-chain fatty acids that can enhance gut health. By slowing the digestion, such resistant starch induces the fullness feelings which help in weight controlling by lowering inclusive calorie intake. V-type of starch also helps in regulating blood sugar levels which is quite beneficial for diabetic patients (Chi et al., 2018).

#### 3.7.6 Water distributions

NMR is a fast and effectual approach employed to enumerate the alterations in water positions and envisage the core water allocation of food (Yang, Cheng, et al., 2021). The preservation of rice grain shape is linked to the water molecules' distributions and proportions. Rice with various moisture distributions results in different tastes (Chen et al., 2021). T_21_, T_22_, and T_23_ relaxation time corresponds to the bound, immobilized, and free water which the inversion spectrum ranges from 0.1 to 10 ms, 10 to 100 ms, and 100 to 1000 ms accordingly. A_21_, A_22_, and A_23_ respectively exhibit the water fractions. The individual values are shown in Table 3 which indicates the interior microstructure was not completely damaged. The fractions T_21_, T_22_, and T_23_ can be associated with the protons of the inner space of the starch-glutelin framework, redeemable protons on the framework & exist in small webbing of cooked rice microstructure, and protons occur at larger interstices or webbing of microstructure (Liu et al., 2023).

**Table 3:** Relaxation times and their corresponding water fractions.

Sample	T_21_ (ms)	A_21_ (%)	T_22_ (ms)	A_22_ (%)	T_23_ (ms)	A_23_ (%)
**Control**	2.95 ± 0.00b	11.42 ± 0.32b	39.45 ± 0.00b	84.70 ± 0.61b	376.37 ± 81.4b	1.79 ± 0.15a
**Optimized**	4.07 ± 0.00a	14.09 ± 0.68a	44.70 ± 3.24a	86.79 ± 0.23a	925.79 ± 0.00a	1.21 ± 0.18b

Initially, starch at the surface was gelatinized during the steaming and dispersed in surplus water. Continuous diffusion of water in grain instigated the structure disruption due to high-pressure steaming and impacted the palatability of steamed rice. Because of water absorbance in grain, starch, and protein formed a 3D microstructure framework. This structure provides ample interplanetary for incongruent proton proportions without aggregating the diffusion. The duration of T2 represents the ease of migration of water molecules i.e., shorter time represents difficulty in migration of water molecules. The control treatment had lower values as compared to the optimized treatment. The elevated values of optimized treatment depict that high-pressure steaming breaks down the microstructure obstacles and reduces the moisture slavery of the innermost steamed rice (Xu et al., 2019).

## 4 Conclusion

The research was conducted to optimize the high-pressure steaming process for japonica rice by employing a three-factor, three-level Box-Behnken design by merging it with particular soaking conditions to improve the rice cooking quality. The optimal conditions achieved were steaming at the temperature of 110 °C and 0.042 MPa with the combination of a soaking ratio of 1:1.3 and a soaking time of 15 min which was found to have a significant impact on textural and gel characteristics that improved the eating quality. The stress relaxation test and pasting curve model highlight the viscoelastic behavior and strong hydrogen bonding respectively. Modeling also depicted the significant impact of soaking conditions on gelatinization properties while the retrogradation phenomenon was elucidated through Ea values. High pressure steaming process also induced a yellowish color due to the Millard reaction whereas rheometer data and microstructural analysis corroborate pseudoplastic behavior and moisture reduction. The current research is based on only one rice variety, various rice varieties and different independent variables can be chosen for further study and various cooking methods can be adopted for the comparison to evaluate the eating qualities of cooked rice.

## Use of AI tool

The authors declare they haven't used any AI tool.

## Funding

The complete research was self-funded.

**Note:** P = Pressure, SR = Soaking ratio, ST = Soaking time, CT = Cooking time.

## CRediT authorship contribution statement

**Saadia Zainab:** Writing – review & editing, Writing – original draft, Software, Data curation, Conceptualization. **Xianqing Zhou:** Validation, Supervision. **Yurong Zhang:** Visualization, Resources. **Saira Tanweer:** Writing – review & editing, Resources, Funding acquisition, Conceptualization. **Tariq Mehmood:** Project administration, Formal analysis.

## Declaration of competing interest

The authors declare that they have no known competing financial interests or personal relationships that could have appeared to influence the work reported in this paper.

## Data Availability

Data will be made available on request.

## Appendix A Supplementary data

Supplementary material Image 1

## References

[bb0005] Aznan A., Viejo C.G., Pang A., Fuentes S. (2023). Review of technology advances to assess rice quality traits and consumer perception. Food Research International.

[bb0010] Bae J.E., Choi Y.J., Kim H.R., Kim Y.S., Choi H.D., Hong J.S. (2023). Impact of steam pressure treatment on rough rice and its physicochemical properties. LWT.

[bb0015] Cai Y., Pan X., Zhang D., Yuan L., Lao F., Wu J. (2024). The kinetic study of 2-acetyl-1-pyrroline accumulation in the model system: An insight into enhancing rice flavor through the Maillard reaction. Food Research International.

[bb0020] Chaturvedi S., Manickavasagan A. (2024). Rice analogues: Processing methods and product quality. Trends in Food Science & Technology.

[bb0025] Chen C., Jiang S., Li M., Li Y., Li H., Zhao F., Pang Z., Liu X. (2021). Effect of high temperature cooking on the quality of rice porridge. International Journal of Agricultural and Biological Engineering.

[bb0030] Chi C., Li X., Feng T., Zeng X., Chen L., Li L. (2018). Improvement in nutritional attributes of Rice starch with dodecyl Gallate complexation: A molecular dynamic simulation and in vitro study. Journal of Agricultural and Food Chemistry.

[bb0035] Devi L.M., Badwaik L.S. (2022). Variety difference in physico-chemical, cooking, textural, pasting and phytochemical properties of pigmented rice. *Food chemistry*. Advances.

[bb0040] Farooq M.A., Murtaza M.A., Aadil R.M., Arshad R., Rahaman A., Siddique R., Haq A.U. (2021). Investigating the structural properties and in vitro digestion of rice flours. Food Science & Nutrition.

[bb0045] Gao Y., Zhang L., Chen W., Zhou W., Deng G., Dai G., Bao J. (2024). Cooked Rice textural properties and starch physicochemical properties from new hybrid Rice and their parents. Foods.

[bb0050] Ha M., Jeong D., Park J., Chung H.J. (2024). Relation between textural attributes and surface leachate structural and compositional characteristics of cooked rice. Food Science and Biotechnology.

[bb0055] Hassan M., Mebarek-Oudina F., Faisal A., Ghaffar A., Ismail A.I. (2022). Thermal energy and mass transport of shear thinning fluid under effects of low to high shear rate viscosity. International Journal of Thermofluids.

[bb0060] He Y., Chen F., Shi Y., Guan Z., Zhang N., Campanella O.H. (2021). Physico-chemical properties and structure of rice cultivars grown in Heilongjiang Province of China. Food Science and Human Wellness.

[bb0065] Hu C., Sha L., Huang C., Luo W., Li B., Huang H., Xu C., Zhang K. (2023). Phase change materials in food: Phase change temperature, environmental friendliness, and systematization. Trends in Food Science & Technology.

[bb0070] Hu H., Li S., Pan D., Wu S., Qiu M., Qiu Z., Liu X., Zhang J. (2022). The variation of Rice quality and relevant starch structure during long-term storage. Agriculture.

[bb0075] Hu W.X., Chen J., Zhao J.W., Chen L., Wang Y.H. (2020). Effect of the addition of modified starch on gelatinization and gelation properties of rice flour. International Journal of Biological Macromolecules.

[bb0080] Kadival A., Mitra J., Kaushal M. (2023). Influence of incorporation of peanut protein isolate on pasting, rheological and textural properties of rice starch. Journal of Food Engineering.

[bb0085] Karimidastjerd A., Gulsunoglu-Konuskan Z., Olum E., Toker O.S. (2024). Evaluation of rheological, textural, and sensory characteristics of optimized vegan rice puddings prepared by various plant-based milks. Food Science & Nutrition.

[bb0090] Kumar R., Singh N., Khatkar B.S. (2023). Effects of A- and B-type starch granules on composition, structural, thermal, morphological, and pasting properties of starches from diverse wheat varieties. Food Bioengineering.

[bb0095] Li H., Gilbert R.G. (2018). Starch molecular structure: The basis for an improved understanding of cooked rice texture. Carbohydrate Polymers.

[bb0100] Li Z., Liang J., Lu L., Liu L., Wang L. (2024). Effect of ferulic acid incorporation on structural, rheological, and digestive properties of hot-extrusion 3D-printed rice starch. International Journal of Biological Macromolecules.

[bb0105] Lina G., Min Z. (2022). Formation and release of cooked rice aroma. Journal of Cereal Science.

[bb0110] Liu Q., Kong Q., Li X., Lin J., Chen H., Bao Q., Yuan Y. (2020). Effect of mild-parboiling treatment on the structure, colour, pasting properties and rheology properties of germinated brown rice. LWT.

[bb0115] Liu X., Chen L., Chen L., Liu D., Liu H., Jiang D., Fu Y., Wang X. (2023). The effect of terminal freezing and thawing on the quality of frozen dough: From the view of water, starch, and protein properties. Foods.

[bb0120] Lu Z., Fang Z., Liu W., Lu D., Wang X., Wang S., Xue J., He X. (2023). Grain quality characteristics analysis and application on breeding of Yuenongsimiao, a high-yielding and disease-resistant rice variety. Scientific Reports.

[bb0125] Ma Z., Zhu Y., Wang Z., Chen X., Cao J., Liu G., Li G., Wei H., Zhang H. (2024). Effect of starch and protein on eating quality of japonica rice in Yangtze River Delta. International Journal of Biological Macromolecules.

[bb0130] Meza S.L.R., Cañizares L., Dannenberg B., Peres B.B., Rodrigues L.A., Mardade C., De Oliveira M. (2024). Sustainable rice bran protein: Composition, extraction, quality properties and applications. Trends in Food Science & Technology.

[bb0135] Na-Nakorn K., Hamaker B.R., Tongta S. (2021). Physicochemical and rheological properties of cooked extruded reformed rice with added protein or fiber. LWT.

[bb0140] Palabiyik İ., Toker O.S., Karaman S., Yildiz Ö. (2017). A modeling approach in the interpretation of starch pasting properties. Journal of Cereal Science.

[bb0145] Ramli H., Zainal N.F.A., Hess M., Chan C.H. (2022). Basic principle and good practices of rheology for polymers for teachers and beginners. Chemistry Teacher International.

[bb0150] Ren X., Qin M., Zhang M., Zhang Y., Wang Z., Liang S. (2022). Highland barley polyphenol delayed the in vitro digestibility of starch and amylose by modifying their structural properties. Nutrients.

[bb0155] Shen Y., He G., Gong W., Shu X., Wu D., Pellegrini N., Fogliano V. (2022). Pre-soaking treatment can improve cooking quality of high-amylose rice while maintaining its low digestibility. Food & Function.

[bb0160] Shi S., Wang E., Li C., Zhou H., Cai M., Cao C., Jiang Y. (2021). Comprehensive evaluation of 17 qualities of 84 types of rice based on principal component analysis. Foods.

[bb0165] Song J., Rong L., Li J., Shen M., Yu Q., Chen Y., Kong J., Xie J. (2024). Effects of three different polysaccharides on the sol gel-behavior, rheological, and structural properties of tapioca starch. International Journal of Biological Macromolecules.

[bb0170] Taghinezhad E., Khoshtaghaza M.H., Minaei S., Suzuki T., Brenner T. (2016). Relationship between degree of starch gelatinization and quality attributes of parboiled Rice during steaming. Rice Science.

[bb0175] Weremfo A., Abassah-Oppong S., Adulley F., Dabie K., Seidu-Larry S. (2022). Response surface methodology as a tool to optimize the extraction of bioactive compounds from plant sources. Journal of the Science of Food and Agriculture.

[bb0180] Witek M., Maciejaszek I., Surówka K. (2020). Impact of enrichment with egg constituents on water status in gluten-free rice pasta-nuclear magnetic resonance and thermogravimetric approach. Food Chemistry.

[bb0185] Xu D., Hong Y., Gu Z., Cheng L., Li Z., Li C. (2019). Effect of high pressure steam on the eating quality of cooked rice. LWT.

[bb0190] Yan W., Yin L., Zhang M., Zhang M., Jia X. (2021). Gelatinization, retrogradation and gel properties of wheat Starch–Wheat bran arabinoxylan complexes. Gels.

[bb0195] Yang H., Cheng S., Lin R., Wang S., Wang H., Wang H., Tan M. (2021). Investigation on moisture migration, microstructure and quality changes of fresh-cut apple during storage. International Journal of Food Science & Technology.

[bb0200] Yang Z., Hao H., Wu Y., Liu Y., Ouyang J. (2021). Influence of moisture and amylose on the physicochemical properties of rice starch during heat treatment. International Journal of Biological Macromolecules.

[bb0205] Yu C., Zhu L., Zhang H., Bi S., Wu G., Qi X., Zhang H., Wang L., Qian H., Zhou L. (2021). Effect of cooking pressure on phenolic compounds, gamma-aminobutyric acid, antioxidant activity and volatile compounds of brown rice. Journal of Cereal Science.

[bb0210] Zainab S., Zhou X., Zhang Y., Tanweer S., Mehmood T. (2024). Suitability of early indica rice for the preparation of rice noodles by its starch properties analysis. Food Chemistry X.

[bb0215] Zhang S., Li Z., Lin L., Zhang L., Wei C. (2019). Starch components, starch properties and appearance quality of opaque kernels from rice mutants. Molecules.

[bb0220] Zhang W., Wang G., Wen P., Chen Y., Yu Q., Shen M., Xie J. (2023). Effect of purple red rice bran anthocyanins on pasting, rheological and gelling properties of rice starch. International Journal of Biological Macromolecules.

[bb0225] Zhang X., Zuo Z., Zhang X., Li T., Wang L. (2024). Pre-gelatinization phenomenon and protein structural changes in rice quality modification by superheated steam treatment. *Food*. Bioscience.

[bb0230] Zhu L., Zhang Y., Wu G., Qi X., Dag D., Kong F., Zhang H. (2020). Characteristics of pasting properties and morphology changes of rice starch and flour under different heating modes. International Journal of Biological Macromolecules.

