# Supplementary material for: Maximizing japonica rice quality by high-pressure steam: Insights into improvement
Source: Food Chem X. 2025 Jan 23;25:102212. doi: 10.1016/j.fochx.2025.102212 (PMC11814526; doi:10.1016/j.fochx.2025.102212)
Supplement: Supplementary file 1 — Supplementary material [file mmc1.docx]

**Figures Captions:**

**Fig. S1.** High-pressure steam process curve

**Fig. S2.** Stress relaxation curves

**Fig. S3.** 3D response surface plots of textural parameters showing the combined effect of steaming conditions and soaking time

**Fig. S4.** 3D response surface plots of pasting parameters showing the combined effect of steaming conditions and soaking time

**Figures:**

**Fig. S1.**


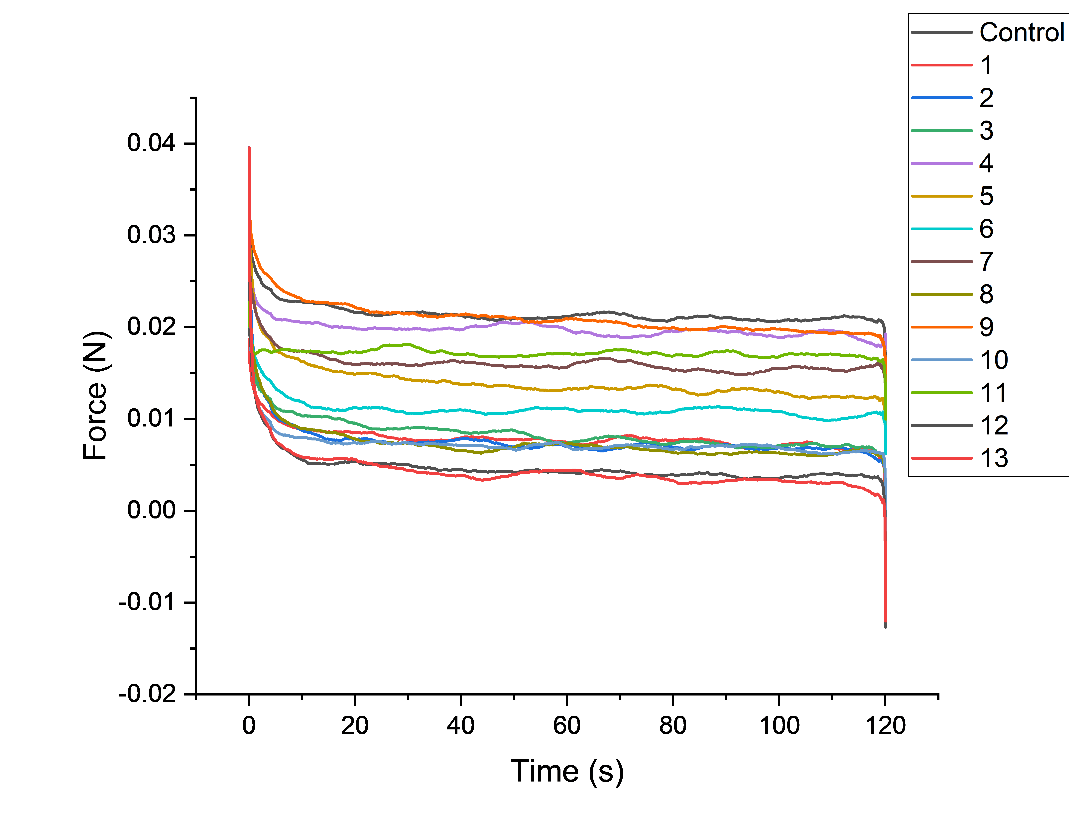


**Fig. S2.**

**
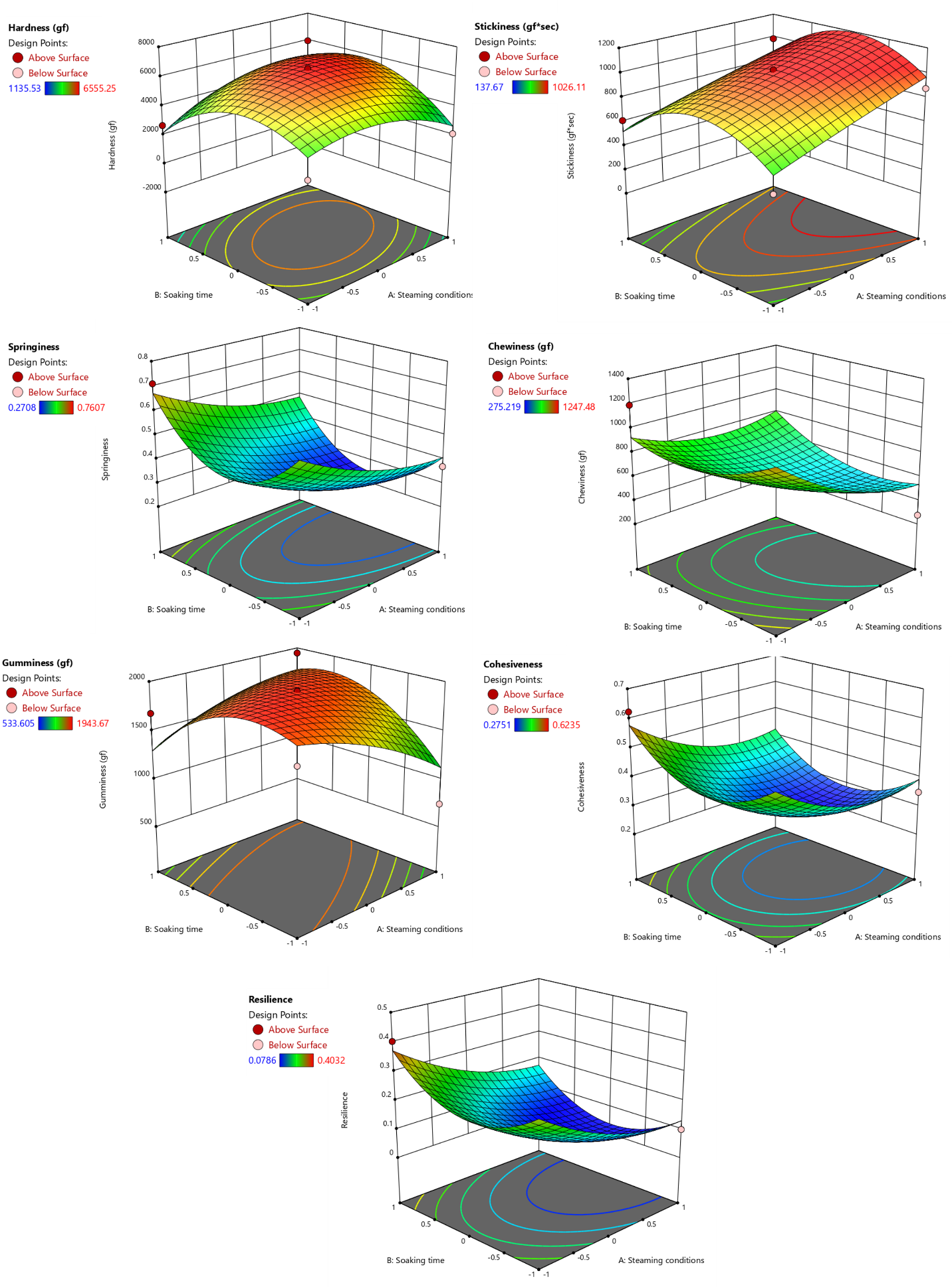
Fig. S3.**

**
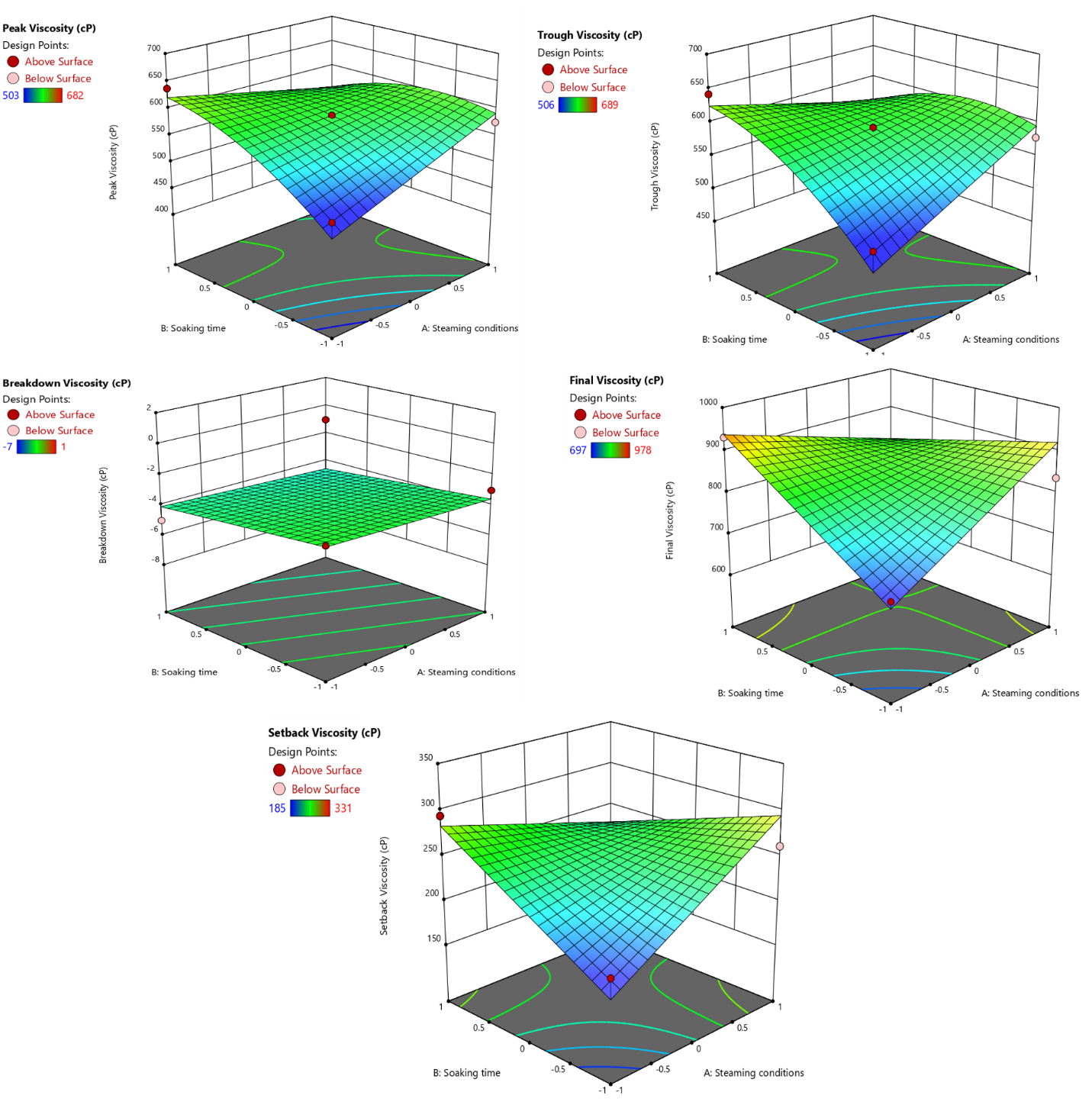
**

**Fig. S4.**

**Tables:**

**Table S1:** Box-Behnken design matrix

| Sr. no. | Variables | Notations |  | Variable Levels | | | |
| --- | --- | --- | --- | --- | --- | --- | --- |
|  |  |  |  | -1 | 0 | | 1 |
| 1 | Steaming Conditions (Temperature: Pressure) (°C: MPa) | X_1_ |  | 110: 0.042 | | 115: 0.069 | 120: 0.097 |
| 2 | Soaking Time (min) | X_2_ |  | 15 | 30 | | 45 |
| 3 | Rice to water ratio | X_3_ |  | 1:1 | 1:1.3 | | 1:1.6 |

**Table S2:** Coded values and real values

| Experimental Run | Coded Values | | |  | Real Values | | | |
| --- | --- | --- | --- | --- | --- | --- | --- | --- |
|  | X_1_ | X_2_ | X_3_ |  | X_1_ (°C: MPa) | | X_2_ (min) | X_3_ |
| Control | - | - | - |  | 100 °C | 30 | | 1:1.3 |
| 1 | 1 | 0 | 1 |  | 120: 0.097 | 30 | | 1:1.6 |
| 2 | 1 | -1 | 0 |  | 120: 0.097 | 15 | | 1:1.3 |
| 3 | 1 | 0 | -1 |  | 120: 0.097 | 30 | | 1:1 |
| 4 | 0 | -1 | 1 |  | 115: 0.069 | 15 | | 1:1.6 |
| 5 | 0 | 1 | 1 |  | 115: 0.069 | 45 | | 1:1.6 |
| 6 | 0 | -1 | -1 |  | 115: 0.069 | 15 | | 1:1 |
| 7 | 0 | 1 | -1 |  | 115: 0.069 | 45 | | 1:1 |
| 8 | -1 | -1 | 0 |  | 110: 0.042 | 15 | | 1:1.3 |
| 9 | -1 | 0 | 1 |  | 110: 0.042 | 30 | | 1:1.6 |
| 10 | 1 | 1 | 0 |  | 120: 0.097 | 45 | | 1:1.3 |
| 11 | -1 | 1 | 0 |  | 110: 0.042 | 45 | | 1:1.3 |
| 12 | -1 | 0 | -1 |  | 110: 0.042 | 30 | | 1:1 |
| 13-17* | 0 | 0 | 0 |  | 115: 0.069 | 30 | | 1:1.3 |

* 5 center points

**Table S3:** Appearance and Physicochemical parameters of rice

| **Appearance Parameters** | |
| --- | --- |
| **Parameter** | **Values** |
| Whole rice rate (%) | 73.6±0.97 |
| Broken rice rate (%) | 26.4±0.97 |
| Yellow grain rice (%) | 1.8±0.05 |
| Chalky grain rate (%) | 45.9±3.02 |
| Length (mm) | 5.2±0.05 |
| Width (mm) | 2.5±0.03 |
| Length / Width | 2.1±0.05 |
| **Physicochemical Properties** | |
| **Parameter** | **Values (% d.b.)** |
| Moisture | 14.02±0.6 |
| Protein | 6.6±0.2 |
| amylose | 18.7±2.6 |
| Fat | 0.35±0.1 |
| Ash | 0.4±0.3 |
| Fiber | 0.8±0.01 |
| Starch | 78.6±0.52 |

Values were taken in triplicates and represented as mean ± SD (standard deviation).

**Table S4:** Textural parameters recorded against each run of experimental design

**Where;** HARD, STICK, CHEW, GUM, SPRING, COH, RES, and GS stand for hardness, stickiness, chewiness, gumminess, springiness, cohesiveness, resilience, and gel strength respectively. Values with dissimilar letters in the same column are significantly different (p<0.05).

| **Samples** | **HARD (gf)** | **STICK (gf*sec)** | **CHEW (gf)** | **GUM (gf)** | **SPRING** | **COH** | **RES** | **GS (g/cm^2^)** |
| --- | --- | --- | --- | --- | --- | --- | --- | --- |
| **Control** | 5541±125c | 1018±27ab | 420±9e | 1480±47cd | 0.28±0.01g | 0.26±0.01f | 0.07±0.01e | 24.53±1.22f |
| **1** | 2003±148g | 800±180d | 448±271d | 807±101f | 0.53±0.25d | 0.40±0.03c | 0.10±0.00d | 43.22±2.16b |
| **2** | 2128±131fg | 875±39cd | 275±26g | 741±32fg | 0.37±0.02e | 0.35±0.01d | 0.1±0.00d | 40.61±2.03c |
| **3** | 3837±205e | 950±16c | 517±29cd | 1420±44e | 0.36±0.03e | 0.37±0.01cd | 0.11±0.01cd | 43.52±2.18b |
| **4** | 1448±187gh | 249±54gh | 428±46de | 638±62gh | 0.67±0.02bc | 0.44±0.02bc | 0.24±0.01c | 31.81±1.60e |
| **5** | 1442±145h | 363±191g | 542±181c | 718±79g | 0.74±0.17ab | 0.50±0.01ab | 0.27±0.03bc | 38.24±1.91cd |
| **6** | 4799±178d | 988±20bc | 548±19bc | 1625±40c | 0.33±0.01f | 0.34±0.01de | 0.11±0.00cd | 38.06±1.90cd |
| **7** | 1135±107j | 211±64h | 346±9f | 533±40i | 0.65±0.05c | 0.47±0.02b | 0.26±0.02bc | 33.51±1.67de |
| **8** | 2631±24f | 460±193f | 1247±183a | 1635±60bc | 0.76±0.08a | 0.62±0.02a | 0.39±0.01a | 41.53±2.07bc |
| **9** | 5202±135cd | 1015±54b | 388±44ef | 1432±100d | 0.27±0.01g | 0.27±0.01f | 0.08±0.00e | 35.81±1.80d |
| **10** | 5963±36b | 983±34bc | 645±61b | 1943±102a | 0.33±0.02f | 0.32±0.01e | 0.10±0.01d | 40.39±2.01c |
| **11** | 2697±345ef | 614±55c | 1189±105ab | 1680±222b | 0.71±0.04b | 0.62±0.03a | 0.40±0.03a | 28.38±1.42ef |
| **12** | 1252±14i | 137±17i | 385±78bc | 624±23h | 0.61±0.10cd | 0.50±0.02ab | 0.30±0.02b | 50.2±2.51a |
| **13** | 6555±156a | 1026±22a | 565±8bc | 1904±37ab | 0.30±0.01g | 0.30±0.01f | 0.08±0.00e | 50.15±2.50a |

**Table S5:** Pasting Parameters recorded against each run of experimental design

| **Samples** | **Peak Viscosity (cP)** | **Trough Viscosity (cP)** | **Final Viscosity (cP)** | **Setback Viscosity (cP)** |
| --- | --- | --- | --- | --- |
| **Control** | 554±38.78h | 559±39.13fg | 582±40.74j | 28±1.96h |
| **1** | 647±45.29bc | 651±45.57b | 978±68.46a | 331±23.17a |
| **2** | 575±40.25f | 578±40.46e | 836±58.52f | 261±18.27d |
| **3** | 682±47.74a | 689±48.23a | 957±66.99b | 275±19.25c |
| **4** | 577±40.39ef | 578±40.46e | 842±58.94ef | 265±18.55cd |
| **5** | 654±45.78b | 658±46.06ab | 915±64.05d | 261±18.27d |
| **6** | 561±39.27g | 564±39.48f | 776±54.32g | 215±15.05ef |
| **7** | 617±43.19d | 621±43.47c | 903±63.21de | 286±20.02bc |
| **8** | 503±35.21j | 506±35.42h | 697±48.79i | 194±13.58f |
| **9** | 618±43.26d | 617±43.19cd | 852±59.64e | 234±16.38e |
| **10** | 528±36.96i | 529±37.03g | 713±49.91h | 185±12.95g |
| **11** | 637±44.59c | 642±44.94bc | 931±65.17c | 294±20.58b |
| **12** | 570±39.9fg | 576±40.32ef | 763±53.41gh | 193±13.51f |
| **13** | 588±41.16e | 593±41.51d | 821±57.47fg | 233±16.31e |

Values with dissimilar letters in the same column are significantly different (p<0.05).

**Table S6:** Eigenvectors of corresponding matrices for rice optimization response variables

| **Parameters** | **PC1** | **PC2** | **PC3** | **PC4** |
| --- | --- | --- | --- | --- |
| Hardness | -0.38751 | 0.02911 | 0.273 | 0.00451 |
| Stickiness | -0.34838 | 0.192 | 0.29183 | 0.06301 |
| Chewiness | 0.09654 | -0.33253 | 0.53774 | 0.09579 |
| Gumminess | -0.26961 | -0.1264 | 0.52935 | 0.0547 |
| Springiness | 0.3849 | -0.22386 | -0.00909 | 0.03575 |
| Cohesiveness | 0.35715 | -0.27282 | 0.17093 | -0.00645 |
| Resilience | 0.32937 | -0.32017 | 0.10837 | -0.04947 |
| Gel strength | -0.11063 | -0.01789 | -0.30395 | -0.09614 |
| Peak | 0.19767 | 0.4441 | 0.16288 | -0.12054 |
| Trough | 0.19977 | 0.44038 | 0.1687 | -0.15352 |
| Breakdown | -0.12179 | -0.0698 | -0.19933 | 0.84039 |
| Final | 0.27484 | 0.37595 | 0.15975 | 0.21942 |
| Setback | 0.28402 | 0.26646 | 0.13138 | 0.4223 |
|  |  |  |  |  |
| Eigenvalue | 5.37 | 3.35 | 1.85 | 1.08 |
|  |  |  |  |  |
| Variance contribution | 41.33 | 25.82 | 14.26 | 8.34 |
|  |  |  |  |  |
| Cumulative contribution | 41.33 | 67.15 | 81.41 | 89.75 |

**Table S7:** Equations of model fit and goodness of fitness of model values

| **Responses** | **Models** | **R^2^** |
| --- | --- | --- |
| **Textural Parameters** | | |
| Hardness | 6555.25+268.68**A**+28.81**B**-115.9**C**+942.1**AB**-1446.27**AC**+914.55**BC**-1166.42**A^2^**-2033.79**B^2^**-2314.91**C^2^** | 0.84 |
| Stickiness | 1026.11+172.97**A**-50**B**+17.58**C**-11.59**AB**-256.76**AC**+222.81**BC**-9.88**A^2^**-282.75**B^2^**-290.16**C^2^** | 0.82 |
| Springiness | 0.2972-0.0942**A**+0.0371**B**+0.0312**C**+0.0028**AB**+0.1288**AC**-0.607**BC**+0.0463**A^2^**+0.1999**B^2^**+0.1033**C^2^** | 0.73 |
| Chewiness | 565.94-165.55**A**+28.13**B**+1.19**C**+107.10**AB**-18.17**AC**+79.13**BC**+121.10**A^2^**+152.44**B^2^**-251.89**C^2^** | 0.70 |
| Gumminess | 1904.70-57.55**A**+29.52**B**-75.79**C**+289.19**AB**-355.39**AC**+292.91**BC**-106.14**A^2^**-298.16**B^2^**-727.44**C^2^** | 0.83 |
| Cohesiveness | 0.2906-0.0714**A**+0.0209**B**-0.0075**C**-0.0062**AB**+0.0640**AC**-0.0190**BC**+0.0693**A^2^**+0.1201**B^2^**+0.0268**C^2^** | 0.72 |
| Resilience | 0.0857-0.0949**A**+0.0255**B**-0.0119**C**-0.0041**AB**-0.0301**BC**+0.0467**A^2^**+0.1151**B^2^**+0.0183**C^2^** | 0.79 |
| **Pasting Parameters** | | |
| Peak Viscosity | 588+13**A**+27.5**B**+8.25**C**-45.25**AB**-20.75**AC**+5.25**BC**-0.1250**A^2^**-27.12**B^2^**+41.37**C^2^** | 0.86 |
| Trough Viscosity | 593+13.25**A**+28**B**+6.75**C**-46.25**AB**-19.75**AC**+5.75**BC**-0.625**A^2^**-28.63**B^2^**+40.88**C^2^** | 0.85 |
| Breakdown Viscosity | -3.82-0.25**A**-0.5**B**+1.5**C** | 0.40 |
| Final Viscosity | 839.29+30.13**A**+38.88**B**+23.50**C**-89.25**AB**-17**AC**-13.5**BC** | 0.60 |
| Setback Viscosity | 244.65+17.13**A**+11.38**B**+15.25**C**-44**AB**+3.75**AC**-18.75**BC** | 0.62 |

Where, **A**: Steaming conditions, **B**: Soaking time, **C**: Soaking ratio

**Table S8:** Power law model parameters

|  |  | **K** | **n** | **R^2^** |
| --- | --- | --- | --- | --- |
| **Strain Sweep** |  |  |  |  |
| **Control** |  | 7.019556 | 0.4758 | 0.9153 |
| **Optimized** |  | 11.3192 | 0.351074 | 0.9002 |
| **Frequency Sweep** |  |  |  |  |
|  |  | **G**$'$ | | |
| **Control** |  | 10.23486 | 0.192557 | 0.9191 |
| **Optimized** |  | 15.97141 | 0.105724 | 0.9134 |
|  |  | **G**$''$ | | |
| **Control** |  | 5.174796 | 0.271631 | 0.9431 |
| **Optimized** |  | 4.74599 | 0.415524 | 0.9353 |
